# Deubiquitinating Enzymes Orchestrate the Cancer Stem Cell-Immunosuppressive Niche Dialogue: New Perspectives and Therapeutic Potential

**DOI:** 10.3389/fcell.2021.680100

**Published:** 2021-06-09

**Authors:** Jun-Nan Guo, Bai-Rong Xia, Shen-Hui Deng, Chang Yang, Ya-Nan Pi, Bin-Bin Cui, Wei-Lin Jin

**Affiliations:** ^1^Department of Colorectal Surgery, Harbin Medical University Cancer Hospital, Harbin, China; ^2^Division of Life Sciences and Medicine, The First Affiliated Hospital of USTC, Anhui Provincial Cancer Hospital, University of Science and Technology of China, Hefei, China; ^3^Department of Anesthesiology, The Fourth Affiliated Hospital of Harbin Medical University, Harbin, China; ^4^Department of Gynecology, Harbin Medical University Cancer Hospital, Harbin, China; ^5^Medical Frontier Innovation Research Center, The First Hospital of Lanzhou University, Institute of Cancer Neuroscience, The First Clinical Medical College of Lanzhou University, Lanzhou, China

**Keywords:** deubiquitylating enzymes, deubiquitination, cancer stem cells, stemness-related signals, inhibitory tumor-associated immune microenvironment

## Abstract

Cancer stem cells (CSCs) are sparks for igniting tumor recurrence and the instigators of low response to immunotherapy and drug resistance. As one of the important components of tumor microenvironment, the tumor associated immune microenvironment (TAIM) is driving force for the heterogeneity, plasticity and evolution of CSCs. CSCs create the inhibitory TAIM (ITAIM) mainly through four stemness-related signals (SRSs), including Notch-nuclear factor-κB axis, Hedgehog, Wnt and signal transducer and activator of transcription. Ubiquitination and deubiquitination in proteins related to the specific stemness of the CSCs have a profound impact on the regulation of ITAIM. In regulating the balance between ubiquitination and deubiquitination, it is crucial for deubiquitinating enzymes (DUBs) to cleave ubiquitin chains from substrates. Ubiquitin-specific peptidases (USPs) comprise the largest family of DUBs. Growing evidence suggests that they play novel functions in contribution of ITAIM, including regulating tumor immunogenicity, activating stem cell factors, upregulating the SRSs, stabilizing anti-inflammatory receptors, and regulating anti-inflammatory cytokines. These overactive or abnormal signaling may dampen antitumor immune responses. The inhibition of USPs could play a regulatory role in SRSs and reversing ITAIM, and also have great potential in improving immune killing ability against tumor cells, including CSCs. In this review, we focus on the USPs involved in CSCs signaling pathways and regulating ITAIM, which are promising therapeutic targets in antitumor therapy.

## Introduction

Cancer cells are heterogeneous, and tumor cells in different states exhibit differences in expression products, immune interactions, tumor proliferation potential, and therapeutic responses (Castagnoli et al., 2020). There are many explanations for these differences, including the cancer stem cell (CSC) hypothesis, which is based on a set of cancer cells with self-renewal ability (Prager et al., 2019). Because CSCs are in an undifferentiated or poorly differentiated state, many stemness-related signals (SRSs) are key to their multiple functions. Some cancers heavily rely on SRSs to promote immunosuppression, metastasis, treatment resistance, and epithelial-mesenchymal transition (Lytle et al., 2018). CSCs can be compared to seeds with the surrounding tumor microenvironment considered the soil in which they grow. CSCs change the tumor microenvironment (TME) through SRSs.

Compared with regulating the entire TME, targeting one microenvironment seems to be a more effective method (Jin and Jin, 2020). As one of the important components of the TME, the tumor- associated immune microenvironment (TAIM) is driving force for the heterogeneity, plasticity and evolution of CSCs. CSCs can create inhibitory TAIMs (ITAIMs) by leveraging the immunomodulatory induction by SRSs (Clara et al., 2020), thereby regulating cytokines and chemokines to strengthen suppressive immune cells, upregulating suppressive immune checkpoints, and downregulating immunogenicity to reduce immune cell recognition and killing (Clara et al., 2020; Ho et al., 2020; Nowak and Klink, 2020). Breakthroughs in immunotherapy have yielded exceptional results in recent years. However, some tumors have a low response to immunotherapy, and the ITAIM is the chief culprit for these poor responses.

Ubiquitin can be linked by one of the seven Lys residues to form ubiquitin chains, including methionine1, K6, K11, K27, k29, k33, K48, and K63 (Wagner et al., 2011). These ubiquitin modifications perform different biological functions, among which K48 and k63 are the most thoroughly studied forms. The K48-linked chains act as a signal of proteasome degradation, and K63-linked chains play an important role in DNA repair (Kaushal and Ramakrishna, 2020). Several studies have documented that K48 and K63 play important roles in regulation of immune responses (Dzimianski et al., 2019). Similar to other post-translational modifications, ubiquitination is reversible. This reversal is achieved by deubiquitylating enzymes (DUBs) in a process called deubiquitination (Mevissen and Komander, 2017). DUBs protect the components of SRSs from degradation by deubiquitination, thus enhancing their reception of SRSs, and directly act on cytokines, inhibitory immune checkpoints or other stem cell factors in the TME. While DUBs activate and upregulate the SRSs, they can also potentially enhance the effect of CSCs on promoting ITAIM, thereby inducing tumor immunity escape, progression and drug resistance (Sun et al., 2020). Recently, DUBs inhibitors have been actively developed, and their therapeutic effects have been confirmed in preclinical trials. However, the space and potential for the exploration of DUBs between CSCs and TAIM regulation remain expansive.

This review summarizes the processes and mechanisms by which CSCs create ITAIMs through four main SRSs. In addition, it summarizes the unique roles of DUBs in regulating stem cell factors, SRSs and TAIMs. The research and application progress of potential target pathways and inhibitors are also reviewed. DUB inhibitors are expected to become CSC-targeted drugs and TAIM modulators in future clinical treatments (Fu et al., 2019; Huang et al., 2019; Dai et al., 2020; Li J. et al., 2020). They can be combined with immunotherapy to enhance the recognition and killing of CSCs to reduce tumor recurrence.

## CSCs and ITAIMs

**FIGURE 1:**
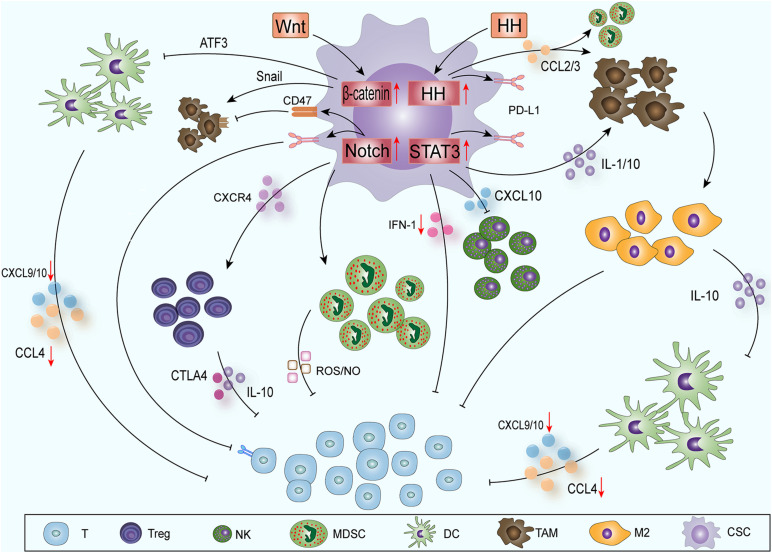
CSCs create ITAIMs mainly through four SRSs: The Notch-NF-κB axis, HH, Wnt and STAT3 pathways. Exogenous Wnt and HH proteins enter CSCs to activate corresponding SRSs. Wnt signaling acts on CD103^+^ DCs by inducing the transcription inhibitor ATF3. The reduction in CCL4, CXCL-9 and -10 reduces CD8^+^ T cell activation and infiltration. HH signaling recruits TAMs and immunosuppresses MDSCs by releasing CCL2 and CCL3. Notch-NF-κB axis signaling mainly regulates Tregs, MDSCs and T cells through a series of cytokines in the TAIM. Activated STAT3 signaling can promote the accumulation of immunosuppressive CD163^+^ TAMs through IL-1 and IL-10. It also downregulates the expression of CXCL-10 and reduces the cytotoxicity of NK cells. The activation of STAT3 inhibits the expression of type I IFN signaling and antitumor immune responses. The HH and Notch-NF-κB axis and STAT3 signaling pathways can also promote PD-L1 expression and suppress the immune response.

CSCs contain some molecular signaling pathways, such as Notch (Grazioli et al., 2017), nuclear factor κB (NF-κB) (Ferrandino et al., 2018), Hedgehog (HH) (Grund-Groschke et al., 2019), Wnt (Luke et al., 2019), signal transducer and activator of transcription (STAT3) (Fouse and Costello, 2013), phosphatase and tensin homolog deleted on chromosome ten (PTEN) (Li et al., 2017) and phosphatidylinositol 3-kinase (PI3K)/protein kinase B (also known as AKT) (Xia and Xu, 2015); these pathways can promote the epithelial-mesenchymal transition, immune cell migration, immunosuppression and treatment resistance. CSCs mainly influence the immune cells around the tumor through the Notch-NF-κB axis, HH, Wnt and STAT3 pathways to create ITAIMs (Figure 1).

### The Notch-NF-κB Axis

Notch is a classic and conservative signaling pathway. The target genes of Notch signaling mainly regulate the phenotype of CSCs, cell survival and differentiation and cell cycle and apoptosis (Andersson et al., 2011). At the same time, Notch is a regulator of CSCs and the TME, and its positive and negative effects have been confirmed by many studies (Weng et al., 2004; Venkatesh et al., 2018). Notch signaling mainly regulates three subgroups in the TAIM: regulatory T cells (Tregs), myeloid-derived suppressor cells (MDSCs) and T cells (Grazioli et al., 2017). In Tregs, Notch can also cascade with NF-κB transcription factors to play a regulatory role. In the TAIM, the classic NF-κB pathway is at the core of Treg functions regulated by Notch1 and Notch3 (Ferrandino et al., 2018). In leukemia, Notch1 can regulate the expression of NF-κB subunits directly to regulate T cells (Osipo et al., 2008) or can bind with the NF-κB subunit to regulate transcription indirectly in different environments and cells (Song L.L. et al., 2008). Notch can also enhance Treg activity through the C-X-C chemokine receptor type 4 (CXCR4) pathway and affect MDSCs through the interleukin (IL) -6-STAT3 axis, thereby promoting the formation of ITAIMs (Weng et al., 2004; Li et al., 2018).

### Hedgehog

HH signaling plays an important role in embryonic development, but its abnormal activation has been shown to be related to the development of a variety of tumors (Katoh, 2019). In recent years, HH signaling has also been found to be related to ITAIM formation and immune escape. Hanna identified that HH signaling alters a critical kinomic signature that enables macrophages to assume the alternative M2 phenotype (Hanna et al., 2019). Chakrabarti’s study showed that HH signaling can induce the expression of programmed death-ligand 1 (PD-L1) in gastric tumor cells and can protect cancer cells from CTL-induced apoptosis (Chakrabarti et al., 2018). It has also been reported that in patients with advanced basal cell carcinoma, the application of HH inhibitors can improve the therapeutic effect of immune checkpoint inhibitors (Nikanjam et al., 2019). In glioma, HH signaling can recruit tumor-associated macrophages (TAMs) and can cause the immunosuppression MDSCs by releasing C-C motif chemokine ligand (CCL) 2/3 (Mi et al., 2020), thereby inducing PD-L1 expression, inducing stromal cells to produce IL10, and promoting the transcription factor forkhead box P3 (FOXP3) expression in Tregs (Grund-Groschke et al., 2019). HH signaling can also be activated by many other intracellular signals, including transforming growth factor β (TGF-β)-, kirsten rat sarcoma viral oncogene (KRAS)-mitogen activated protein kinase (MAPK)/extracellular regulated protein kinases (ERK)-, PI3K- AKT-, insulin-like growth factor—and tumor necrosis factor (TNF) -α induced mammalian target of rapamycin (mTOR)/ribosomal S6 kinase 1 (S6K1) activation (Takebe et al., 2015; Giroux-Leprieur et al., 2018).

### Wnt

With the advancement of sequencing technology and the comprehensive understanding of cancer genomes, abnormal Wnt signaling has been observed in many cancer entities (Ruan et al., 2020; Yang et al., 2020). In the past few years, three types of Wnt cascade signaling have been described. Among these pathways, the typical Wnt signaling pathways related to tumors involves the activation of the T-cell-specific β-catenin–transcription factor–lymphoid enhancer-binding factor pathway, which plays a key role in immune regulation (Zhong and Virshup, 2020). The Wnt/β-catenin pathway has been identified as one of the important signaling pathways related to immune escape. In creating ITAIMs, Wnt signaling acts Wnt signaling on CD103^+^ dendritic cells (DCs) by inducing the expression of the transcription inhibitor ATF3, reducing the production of CCL4, C-X-C motif chemokine ligand (CXCL) -9 and -10 and inhibiting the activation and infiltration of CD8^+^ T cells (Fu et al., 2015). At the same time, in a pan-cancer analysis based on TCGA database information, Wnt/β-catenin pathway activation is usually associated with low T-cell infiltration and immune rejection (Luke et al., 2019). β-catenin signaling activation reduces the recruitment of DCs in hepatocellular carcinoma, thereby impairing T-cell activity (Ruiz de Galarreta et al., 2019). β-catenin signaling can also inhibit innate immunity by regulating interferon regulatory factor (IRF) 3, thereby downregulating the production of type I interferon (IFN) in colorectal cancer (Ding et al., 2018). Wnt signaling also affects TAMs through Snail to produce IL-1β and increase β-catenin activity (Spranger and Gajewski, 2015) to maintain Wnt signaling activation (Boulter et al., 2015).

### STAT3

The activation of STAT3 signaling in CSCs is key to tumor progression because it promotes immunosuppressive factors and angiogenesis (Sasidharan Nair et al., 2018; Wang Y. et al., 2018). Many oncogenic signaling pathways, such as RAS/BRAF/MEK, can participate in the regulatory network of a TAIM. They cooperate with STAT3 and NF-κB signaling to regulate the expression of cytokines [IL-6, IL-1, IL-10, TNF, and vascular endothelial growth factors (VEGFs)] (Sasidharan Nair et al., 2018; Ji et al., 2019). Activated STAT3 signaling can promote the accumulation of immunosuppressive CD163^+^ TAMs and has an inhibitory effect on T cells. Depletion of CD163^+^ TAMs can enhance the infiltration of CD8^+^ T cells into melanoma and promote CD8^+^ T-cell-mediated tumor regression in mice (Etzerodt et al., 2019). T helper cell (Th) 2 and 17, Tregs and MDSCs produce inflammatory cytokines such as IL-1, IL-17, IL-10, TGF-β, and VEGFs through STAT3 signaling. These cytokines can promote the M2 polarization of macrophages and the proliferation of MDSCs, accelerating the formation of ITAIMs and the proliferation of tumor cells (Owen et al., 2019). The activation of STAT3 has been shown to inhibit the expression of STAT1, IRF7 and IRF 9 genes, type I IFN signaling and antitumor immune responses (Lu et al., 2018). Loannis confirmed that STAT3 signaling promotes PD-L1 expression and suppresses the immune response in breast cancer. In addition, STAT3 downregulates the expression of CXCL-10 and reduces the cytotoxicity induced by natural killer cells (NK) (Clark et al., 2019).

## DUBs Involved in Stem Cell Factors, SRSs and ITAIM

Ubiquitination and deubiquitination constitute a whole, dynamic and specific biological process (Wang and Le, 2019). They are involved in the regulation of almost all life activities (Clague et al., 2019). Even in the progression and immune escape of a variety of cancer cells, DUBs also play novel functions, including regulating tumor immunogenicity, activating stem cell factors, upregulating the SRSs related to CSCs, stabilizing anti-inflammatory receptors, and regulating anti-inflammatory cytokines (Zhang et al., 2008; Qiu et al., 2017; Wang Y.C. et al., 2018; Huang et al., 2019; Lei et al., 2019; Li L. et al., 2019; Lai et al., 2020; Li J. et al., 2020). Different studies have emphasized the mechanisms of CSCs in the “re-editing” of the immune system to change the balance between antitumor immune cells and tumor-promoting immune cells. Research on DUBs that can regulate CSCs and ITAIMs has great potential for the use of synergistic immunotherapy. The therapeutic effects of DUB inhibitors such as USP1, USP4, USP7, USP14 and USP33 have been confirmed in prostate cancer, lung cancer, breast cancer and hematological malignancies (Ma et al., 2019; Xia et al., 2019a; Guo et al., 2020; Gutierrez-Diaz et al., 2020; Lai et al., 2020).

**FIGURE 2:**
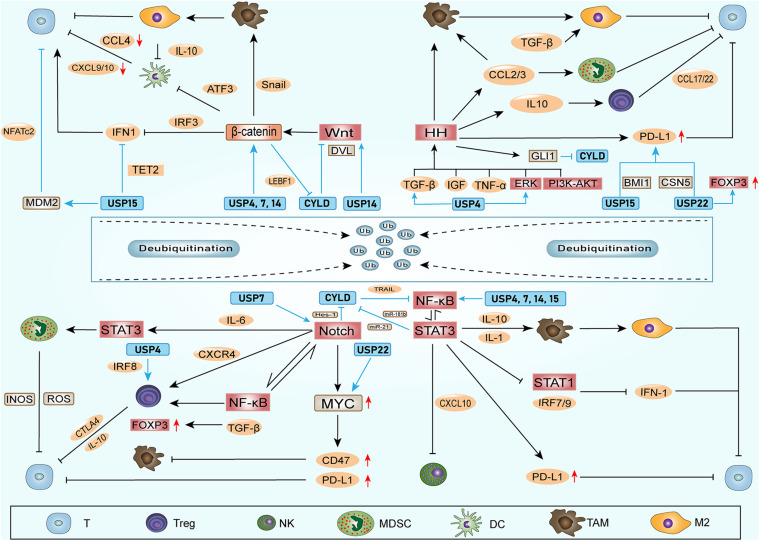
DUBs involved in stem cell factors, SRSs and ITAIM. DUBs can regulate the stem cell factors and the components of SRSs, promoting stemness of CSCs. Additionally, DUBs can affect ITAIMs directly by acting on cytokines, inhibitory immune checkpoints or other oncogenes. The combination of DUBs with substrates leads to deubiquitination and protects substrates from degradation. Therefore, the protein level of the substrate and the signaling pathways involved are upregulated. The arrow at the end of the line in the figure represents upregulation, and the horizontal line represents downregulation.

Ubiquitin-specific peptidases (USPs) comprise the largest family of deubiquitinases. Any abnormal immune response activation will cause pathological damage; therefore, the immune response is also strictly regulated. Among these regulators, DUBs in the ubiquitin-proteasome system play vital roles in regulating the immune response (Lopez-Castejon and Edelmann, 2016). Without exception, DUBs can directly affect the ITAIM by acting on cytokines and inhibitory immune checkpoints. In addition, nearly one-half of the USPs that have been identified can exhibit potential carcinogenic and cancer-promoting functions (Cheng et al., 2019). Among these USPs, USP4, USP7, USP14, USP15, USP22, and CYLD have been the most widely studied, and they could also regulate the stem cell factors and SRSs (Table 1 and Figure 2).

**TABLE 1:** The DUBs associated with stem cell factors and the stemness-related signals.

**DUBs**	**Tumor**	**Substrates**	**Stemness-related signals**	**References**
USP4	Colon cancer	β-catenin	Wnt/β-catenin	Nguyen et al., 2019
USP4	Liver cancer	TGFβR-1	HH	Qiu C. et al., 2018
USP4	Glioblastoma	TGFβR-1	ERK˴HH	Zhou et al., 2019
USP4	Lung adenocarcinoma	β-catenin	Wnt/β-catenin	Hwang et al., 2016
USP4		IRF8		Lin et al., 2017
USP7	T-Cell Leukemia	Notch1	Notch	Jin et al., 2019
USP7	Colon cancer	β-catenin	Wnt/β-catenin	Novellasdemunt et al., 2017
USP7	Multiple myeloma	NEK2	NF-κB˴Notch	Franqui-Machin et al., 2018
USP7	Promyelocytic leukemia protein	PTEN	PTEN/PI3K/AKT mTOR	Song M.S. et al., 2008
USP7		FOXP3	FOXP3	van Loosdregt et al., 2013
USP14	Lung adenocarcinoma	β-catenin	Wnt/β-catenin	Wu et al., 2013
USP14	Colon cancer	DVL	Wnt	Jung et al., 2013
USP15	Glioblastoma	TGFβR-1	HH	Eichhorn et al., 2012
USP15		TAB2 and TAB3	Notch/NF-κB	Zhou Q. et al., 2020
USP15	Glioblastoma	TGFβR-1	HH	Eichhorn et al., 2012
USP15		MDM2	MDM2	Zou et al., 2014
USP15	Malignant Hematopoiesis	MDM2	MDM2	Niederkorn et al., 2020
USP15	Melanoma	TET2		Chen L.L. et al., 2020
USP15	Breast cancer	BMI1	BMI1	Zhang L. et al., 2020
USP22	Non-small cell lung cancer	PD-L1		Wang et al., 2020
USP22	Liver cancer	PD-L1		Huang X. et al., 2020
USP22	Gastric cancer	SOS1	PI3K/AKT	Lim et al., 2020
USP22	Glioblastoma	BMI1	BMI1	Qiu et al., 2020
USP22	Colon cancer	BMI1	BMI1	Yuan et al., 2019
USP22	Gastric cancer	MYC	c-Myc/NAMPT/SIRT1	Liu H. et al., 2019
USP22		FOXP3	FOXP3	Cortez et al., 2020
USP22		MED1		Zhang Y. et al., 2020
CYLD	Cylindroma skin tumors	DVL	Wnt/β-catenin	van Andel et al., 2017
CYLD	Chronic myeloid leukemia	DVL	Wnt/β-catenin	Caliskan et al., 2017
CYLD	Cervical carcinoma	TRAF˴Bcl-3	Notch/NF-κB	Brummelkamp et al., 2003
CYLD	Skin tumor	TRAF˴Bcl-3	Notch/NF-κB	Massoumi et al., 2006
CYLD	Salivary gland tumor	NF-κB	Notch/NF-κB	Fukuda et al., 2008
CYLD	Breast cancer	NF-κB	Notch/NF-κB	Hayashi et al., 2014
CYLD	Hepatocellular carcinoma	TRAIL	Notch/NF-κB	Chu et al., 2006

*DUBs, deubiquitylating enzymes; CSCs, cancer stem cells; USP, ubiquitin-specific peptidase; TGFβR-1, TGF-β receptors; HH, hedgehog; ERK, extracellular regulated protein kinases; IRF, interferon regulatory factor; NF-κB, nuclear factor κB; PI3K, phosphatidylinositol 3-kinase; AKT, protein kinase B; mTOR, mammalian target of rapamycin; FOXP3, forkhead box P3; PTEN, phosphatase and tensin homolog deleted on chromosome ten; DVL, disheveled; PD-L1, programmed death-ligand 1; SOS1, son of sevenless 1; NAMPT, Nicotinamide phosphoribosyltransferase; TRAIL, TNF-related apoptosis-inducing ligand.*

### USP4

USP4 not only plays a role in promoting cancer in a variety of tumor tissues but also indicates a poor prognosis when highly expressed (Yun et al., 2015; Cao et al., 2016; Qiu C. et al., 2018). USP4 can promote cancer in various ways, such as inhibiting SMAD4 monoubiquitination and promoting activin and BMP signaling (Zhou et al., 2017), promoting breast cancer metastasis via the p21-activated kinase 5-aspartyl aminopeptidase-USP4 axis (Geng et al., 2020) and promoting the invasion of breast cancer cells via the relaxin/TGF-β1/SMAD2/MMP-9 signaling pathway (Cao et al., 2016). In recent studies, we found that the USP4 are also involved in the regulation of the components in SRSs. USP4 promotes liver cancer metastasis by upregulating TGF-β signaling to induce the epithelial-mesenchymal transition (Qiu C. et al., 2018) and activate the ERK pathway by regulating TGF-β to promote the development of glioblastoma (Zhou et al., 2019). Both TGF-β and ERK signaling can activate HH signaling in CSCs. USP4 also stabilize β-catenin in brain metastasis lung adenocarcinoma to affect metastasis ability (Hwang et al., 2016). In colorectal cancer, USP4 upregulates Wnt/β-catenin signaling through deubiquitination and facilitates the nuclear localization of β-catenin (Yun et al., 2015; Nguyen et al., 2019). Recent studies have found that USP4 can deubiquitinate the transcriptional factor retinoic acid-related orphan receptor γt in an inflammatory environment and play a positive regulatory role in Th17 cell function (Yang et al., 2015). Th17 cells can produce inhibitory inflammatory cytokines through STAT3 signaling. In addition, USP4 stabilizes IRF8 through K48-linked deubiquitination and enhances the immunosuppressive function of Tregs (Lin et al., 2017). In summary, USP4 can strengthen the SRSs and directly act on immune cells to promote the ITAIM.

### USP7

USP7 has been found to be highly expressed in a variety of tumors and is associated with poor prognosis (Zhang et al., 2016; Wang X. et al., 2018; Xia et al., 2019b; Zhang M.J. et al., 2019). The regulatory role of USP7 in TAIMs has attracted increasing attention, and it can also affect the SRSs. In T-cell leukemia, USP7 can regulate Notch1 at the transcriptional and post-translational levels and lead to increased expression of Notch1 target genes (Shan et al., 2018; Jin et al., 2019). After inhibiting USP7, Notch1 is ubiquitinated and degraded (Jin et al., 2019). In colorectal cancer, USP7 activates Wnt signaling through β-catenin deubiquitination to promote the growth of ectopic transplantation tumors (Novellasdemunt et al., 2017). It can also prevent the ubiquitination of NF-κB and its regulator NEK2 (Colleran et al., 2013; Franqui-Machin et al., 2018), activating NF-κB signaling. In addition, monoubiquitinated PTEN can be deubiquitinated by USP7, leading to the elimination of PTEN in the nucleus and activating the PI3K/AKT/mTOR signaling pathway. PTEN/PI3K/AKT, as one of the SRS pathways of CSCs, has been proven to maintain stemness and promote tumor growth in a variety of cancers (Li et al., 2017; Hu et al., 2019; Luongo et al., 2019). Classic PTEN/PI3K/AKT signaling plays a core role in maintaining the characteristics and stability of Tregs (Brandmaier et al., 2017). USP7 can also reduce and stabilize the ubiquitination of FOXP3 directly, enhancing the immunosuppressive ability of Tregs (van Loosdregt et al., 2013). Tregs suppress the immune response by expressing cytotoxic T-lymphocyte-associated protein 4 (CTLA-4), IL-10, TGFβ, etc. This immunosuppressive property promotes the immune escape of tumor cells (Li C. et al., 2020). In addition, immunosuppressive PD-1 signaling prevents CK2-mediated degradation of PTEN by reducing the expression of casein kinase 2 (CK2) (Patsoukis et al., 2013). In summary, USP7 can directly upregulate the SRSs of multiple cancers and enhance the effect of immunosuppressive cells.

### USP14

USP14 has been extensively studied because of its overexpression in lung cancer (Han et al., 2019), breast cancer (Xia et al., 2019a), ovarian cancer (Luo et al., 2019), and oesophageal squamous cell carcinoma (Sha et al., 2019). It may promote tumorigenesis in approximately 61% of cancers (Liu B. et al., 2019). USP14 inhibitors have also shown treatment efficacy in research settings (Xia et al., 2019a). We focus on the mechanism of USP14 involved in the regulation of SRS and ITAIM. In terms of WNT/β-catenin signaling, USP14 is overexpressed in lung adenocarcinoma and promotes cancer cell proliferation by upregulating β-catenin (Wu et al., 2013). Another study found that USP14 deubiquitinates the k63-linked polyubiquitin and dissociates from DVL. The DVL is subsequently phosphorylated and then activates and upregulates Wnt signaling (Jung et al., 2013). USP14 also promotes cell adhesion-mediated drug resistance by enhancing Wnt signaling in multiple myeloma cells (Xu et al., 2017). It has been reported that the inflammation regulation mediated by USP14 through lipopolysaccharides is related to the activation of NF-κB (Liu et al., 2017). Furthermore, USP14 also accelerates the degradation of IκBα mediated by the proteasome to promote the activation of NF-κB (Li M. et al., 2019). In addition, Xu found that USP14 cause resistance to cisplatin through the activation of AKT signaling in gastric cancer (Fu et al., 2018). In summary, the research on USP14 has made great progress in delineating its roles in the proliferation, migration and autophagy of different cancer cells. Although USP14 inhibitors have also been shown to be useful in cancer treatment, broad space for research on USP14 with respect to ITAIM regulation remains open because of its activation and upregulation of SRS.

### USP22

USP22 was thought to have a cancer-promoting effect, and its gene was confirmed as one of 11 genes called “death-from-cancer” (Glinsky et al., 2005). However, in recent studies, the cancer-promoting or anticancer effects of USP22 were found to depend mainly on its environment (Jeusset and McManus, 2017). In most tumors, high expression of USP22 predicts a poor prognosis (Hu et al., 2015; Lim et al., 2020), and USP22 also been shown to promote tumor proliferation, invasion and drug resistance through a variety of substrates, including PD-L1 (Huang et al., 2019; Huang X. et al., 2020; Wang et al., 2020), RAS activator son of sevenless 1 (SOS1) (Lim et al., 2020), BMI1 (Ma et al., 2017; Qiu G.Z. et al., 2018; Yuan et al., 2019; Qiu et al., 2020), MYC (Liu H. et al., 2019; Zhang K. et al., 2019), and COX-2 (Xiao et al., 2015). These substrates include inhibitory immune checkpoints, transcriptional activators and oncogenes. In non-small cell lung cancer, USP22 can directly deubiquitinate PD-L1 and regulate the level of PD-L1 through the USP22-CSN5-PD-L1 axis, resulting in decreased T-cell proliferation and activation (Wang et al., 2020). Furthermore, studies have shown that knocking out USP22 inhibits the growth of liver cancer in an immune-dependent manner. Tumor immunogenicity and lymphocyte infiltration were enhanced, and the efficacy of PD-L1 immunotherapy and CDDP were also improved (Huang et al., 2019). SOS1, another substrate of USP22, was found to activate RAS signaling and upregulate the PI3K/AKT pathway (Lim et al., 2020). The PI3K/AKT pathway is a carrier of SRSs and promotes Treg activation (Brandmaier et al., 2017). The stem cell factors BMI1 has been proven to be a downstream target of USP22 in glioma, gastric and colorectal cancer. It is upregulated by USP22 deubiquitination (Ma et al., 2017; Qiu G.Z. et al., 2018; Yuan et al., 2019; Qiu et al., 2020). The latest research found that inhibiting BMI1 can lead to the recruitment and activation of CD8^+^ T cells by stimulating IRF3. Targeting BMI1 may eliminate CSCs by silencing PD-L1, and BMI1 inhibition can also attenuate tumor metastasis and recurrence by activating endogenous immunity (Jia et al., 2020). Therefore, USP22 may create ITAIMs through the BMI1 signaling pathway. USP22 can exert its cancer-promoting effect by upregulating the nicotinamide phosphoribosyltransferase (NAMPT)/SIRT1, AKT and ERK signaling pathways involved with c-Myc activation (Liu H. et al., 2019; Zhang K. et al., 2019). In a report, Myc upregulated the expression of CD47 and PD-L1 on the surface of CSCs. CD47 inhibited phagocytosis by binding to sirpα on macrophages (Park and Surh, 2017). In a study using CRISPR to screen the ubiquitination modulators of FOXP3 in Tregs, it was found that USP22 and Atxn713, members of the deubiquitination module of the SAGA stain complex, were positive regulators of FOXP3. In other words, USP22 may inhibit the immune response to tumor cells by directly acting on FOXP3 (Cortez et al., 2020). Furthermore, Han also proved that USP22 can promote tumor development through deubiquitination and immunosuppression in lung adenocarcinoma (Han et al., 2020). USP22 was found to activate MED1 transcriptionally to regulate NK cell development through histone H2A deubiquitination (Zhang Y. et al., 2020).

In summary, the regulation of USP22 in CSCs and TAIMs is abundant and has the potential to suppress immunity in many ways. In some preclinical experiments, USP22 used as a regulator achieved encouraging effects (Li J. et al., 2020).

### USP15

USP15 has a high degree of homology with the abovementioned carcinogenic USP4, and its gene upregulation has been found in glioblastoma, breast cancer and ovarian cancer (Chou et al., 2017). However, USP15 is the same as USP22 in that its role in cancer depends on the environment (Cheng et al., 2019). USP15 acts as a regulator of the natural immune response in the anti-infection processes of the human body (Torre et al., 2017; Kusakabe et al., 2019; Huang L. et al., 2020). It has been reported that USP15 can participate in regulating multiple stem cell factors, including MDM2 (Zou et al., 2014; Niederkorn et al., 2020), TET2 (Chen L.L. et al., 2020), BMI1 (Zhang L. et al., 2020), NF-κB (Zhou Q. et al., 2020), and TGF-β (Eichhorn et al., 2012). The aforementioned signaling pathways activated by these factors can promote the production of IATIM. MDM2 not only can promote tumor progression by downregulating P53 (Niederkorn et al., 2020) but can also negatively regulate Tregs by acting on the transcription factor NFATc2 (Zou et al., 2014). In breast cancer, USP15 is found to deubiquitinate and stabilize BMI1 protein at lys-81 (Zhang L. et al., 2020). Recent studies have also found that USP15 exerts a deubiquitination effect at the K1299 site of TET2 and negatively regulates the activity of TET2. Therefore, USP15 weakens the TET2-mediated IFN/JAK/STAT pathway and reduces the expression of chemokines and lymphocytic infiltration. Knocking out USP15 improves the response of mice with transplanted melanoma to immunotherapy and prolongs survival (Chen L.L. et al., 2020). In summary, USP15 mainly acts on stem cell factors to affect CSCs and ITAIM, other potential pathways need to be explored and verified.

### CYLD

CYLD plays a major role in cell development and proliferation, and it is also an important inflammation regulator (Hadian et al., 2011). CYLD inhibits the NF-κB pathway by deubiquitinating key factors, including NF-κB essential modulators IKKg and TRAF2 (Kovalenko et al., 2003). In contrast to other oncogenic DUBs of the USP family, CYLD is the DUB with the most typical tumor suppressor effects. Patients with high CYLD expression and multiple myeloma (van Andel et al., 2017), oral squamous cell carcinoma (Shinriki et al., 2018) or breast cancer (Hayashi et al., 2014) have a better prognosis. Several substrates of CYLD have been identified in recent studies, including DVL (Tauriello et al., 2010), TRAF (Brummelkamp et al., 2003), Bcl-3 (Massoumi et al., 2006), IKK (Chu et al., 2006), and p53 (Fernandez-Majada et al., 2016). Some of these substrates act as CSCs factors, which can activate and upregulate the SRSs. For example, the upregulation of CYLD causes DVL deubiquitination, significantly inhibiting the oncogenic Wnt pathway, thereby inhibiting the occurrence of tumors (Caliskan et al., 2017). The downregulation of CYLD promotes the accumulation of β-catenin in the nucleus through ubiquitination of DVL at Lys-63 (Tauriello et al., 2010). In addition, CYLD deubiquitinates TRAF and Bcl-3 proteins directly to downregulate NF-kB activity, inhibiting oncogenic transformation in keratinocytes (Brummelkamp et al., 2003; Massoumi et al., 2006). In salivary gland tumors, CYLD has a negative regulatory effect on NF-kB activity after TNF-α stimulation (Fukuda et al., 2008). Studies have also found that the downregulation of CYLD can promote breast cancer metastasis by activating NF-κB (Hayashi et al., 2014). In the inhibition process of TNF-related apoptosis-inducing ligand (TRAIL)-mediated NF-κB, the direct combination of CYLD and IKK enhances the effect of TRAIL (Chu et al., 2006).

The SRSs and CYLD influence each other. The upstream regulatory mechanism of CYLD transcription and translation has been extensively studied in tumors. A variety of oncogenic signaling pathways can regulate CYLD, and some of them belong to SRSs. Studies have shown that Hes-1, a downstream factor of Notch in T-cell leukemia, can reduce the expression of CYLD (D’Altri et al., 2011). STAT3 signaling can activate miR-21 (Iliopoulos et al., 2010) and miR-181b (Xu et al., 2016) to inhibit CYLD transcription. In chronic leukemia, lymphoid enhancer binding factor 1 (LEBF1) downregulates CYLD at the transcriptional level through the WNT/β-catenin signaling pathway (Liu et al., 2012). HH signaling in basal cell carcinoma downregulates the expression of CYLD through GLI1 under the action of the transcription repressor Snail (Kuphal et al., 2011).

In summary, CYLD, as a tumor-suppressing DUB, has a close relationship with CSCs. It potentially reverses the ITAIM by regulating the SRSs, and even improve the effect of immunotherapy.

## Recurrence Caused by CSCs After Initial Treatment

Although surgery, radiotherapy, chemotherapy, and immunotherapy have been individualized and combined clinically, they still cannot completely solve the problems of tumor recurrence and treatment resistance (Das and Law, 2018). The initial comprehensive clinical treatment can eliminate most tumor cells, but CSCs can evade the killing of immune cells by creating ITAIMs, reducing their autoantigenicity, and upregulating inhibitory immune checkpoints. CSCs can also resist radiotherapy and chemotherapy through protective autophagy, efficient cell cycling, promotion of the epithelial-mesenchymal transition, removal of reactive oxygen species, drug efflux, DNA repair, and interactions with the TME (Najafi et al., 2019). These CSCs survive and may remain quiescent for a long time without losing their tumorigenic potential (Quayle et al., 2018; Brown et al., 2019). They are temporarily in balance with the immune system. However, due to aging, immunosuppressive therapy, disease or other factors, the immune system may lose its ability to suppress these CSCs, leading to refractory tumor recurrence (Bruttel and Wischhusen, 2014). As described above, CSCs act as tumor seeds. If they cannot be eradicated during the initial treatment, they will grow like weeds. A prairie fire cannot not destroy them; they shoot up again when the spring breezes blow.

In traditional treatment, most of the visible tumor tissue is removed by surgery, but this treatment creates a hypoxic environment that is beneficial to the maintenance of CSC stemness. In addition, lymph node dissection destroys the optimal microenvironment for T-cell activation (Najafi et al., 2020). Radiotherapy can only kill local tumor cells. Although chemotherapy can act on tumor cells in the blood, it cannot kill CSCs. Moreover, some cytotoxic drugs kill immune effector cells directly or indirectly, resulting in immune tolerance, suppression or incompetence (Shaked, 2016). Therefore, traditional treatment not only fails to prevent treatment failure and death caused by drug resistance and refractory recurrence but also destroys the immune system, which is not conducive to immunotherapy.

However, initial treatment can block the direct and indirect regulation pathways of ITAIM and reverse it. The purpose is to enhance the recognition and killing of CSCs by immunotherapy. Relying on autoimmune function to eliminate residual tumors, including CSCs, is the most promising way to prevent tumor recurrence.

## Research on DUB Inhibitors and their Effects on SRSs and TAIM

**FIGURE 3:**
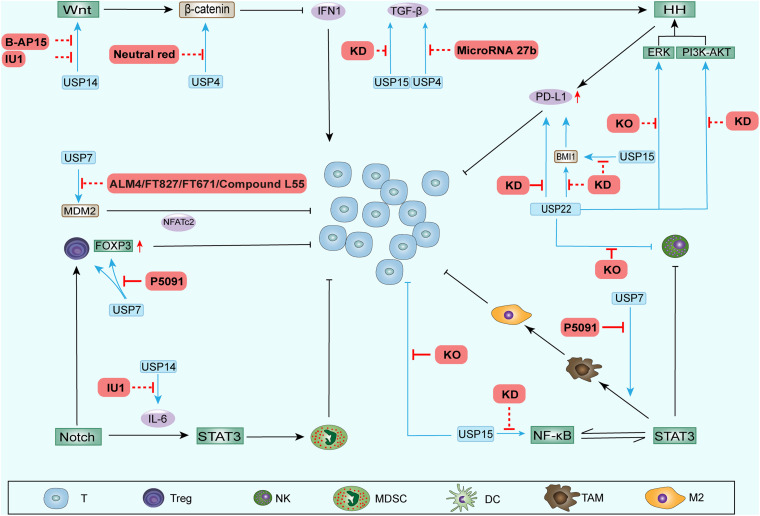
DUB inhibitors and other inhibition methods performed to regulate the TAIM. Some inhibitors and other inhibition methods can block the upregulation of stemness-related signaling pathway components by DUBs. These processes weaken the function of CSCs during ITAIM creation through SRSs and possibly reverse the ITAIM. In addition, knocking out or knocking down some DUB genes can directly regulate the recruitment, quantity and function of immune cells in the TAIM. The red solid line represents the regulatory effect in the TAIM detected in preclinical trials, and the red dotted line represents the potential regulatory effect.

Some DUB inhibitors have been actively developed and play inhibitory roles in tumor development. We summarize the recent progress in inhibitors of DUBs reported above (Table 2 and Figure 3).

**TABLE 2:** Related research on inhibitors of DUBs that regulate TAIM.

**Regulate TAIM**	**DUBs inhibitors**	**DUBs**	**Selectivity**	**Function**	**References**
Potential	Neutral red	USP4	HS	Suppresses colorectal cancer by regulating β-Catenin signaling	Nguyen et al., 2019
Potential	MicroRNA 27b	USP4	HS	Protect Hepatocytes From TGF-β	Wu et al., 2019
Potential	ALM4	USP7	HS	Increases p53 and decreases MDM2 total levels in cells	Gavory et al., 2018
Potential	FT827	USP7	HS	Degradation of oncogenic E3 ligase MDM2 to inhibit tumor	Turnbull et al., 2017
Potential	FT671	USP7	HS	Degradation of oncogenic E3 ligase MDM2 to inhibit tumor	Turnbull et al., 2017
Detected	P5091	USP7	HS	Reduce the proportion of Treg cells	Fu et al., 2019
Detected	P5091	USP7	HS	Impair Treg cell function and promotes antitumor immunity	Wang et al., 2016
Detected	P5091	USP7	HS	Reprogram TAM to regulate antitumor immune response	Dai et al., 2020
Potential	Compound L55	USP7	HS	Antitumor by inducing cell death and reduce the levels of MDM2	Li M. et al., 2020
Potential	IU1	USP14	PS	Decrease TNF-α, IL-1-β, IL-6 and IL-8, and increase I-κB	Kiprowska et al., 2017
Potential	IU1	USP14	PS	Down regulation of Wnt/β- Catenin and PI3K/AKT pathway	Xia et al., 2019a
Potential	B-AP15	USP14	PS	Inhibition of Wnt /β- Catenin and TGFβ/ Smad pathway	Jiang et al., 2019

*ND, not detected; HS, highly selective; PS poor selectivity.*

As one of the most extensively studied inhibitors, USP7 inhibitors have played an effective and highly specific role in many studies. They have a significant inhibitory effect on tumors (Lee et al., 2020; Leger et al., 2020; Ohol et al., 2020; Schauer et al., 2020a). P22077/50429 exerted anti-oncogene function in a variety of malignant tumors (Fan et al., 2013; Zhang W. et al., 2020) and improved chemoresistance in pancreatic cancer (Chen H. et al., 2020). FT827/671 inhibited tumors by degrading the oncogenic E3 ligase MDM2 (Turnbull et al., 2017). In the latest study, P5091 inhibitors showed robust tumor suppression effects in preclinical models of various tumors (An et al., 2017; Wang et al., 2017; Hayal et al., 2020; He et al., 2020; Lee et al., 2020). Among these cancers, in colorectal cancer, P5091 improves the antitumor immune response and reduces the proportion of Tregs in tumor xenografts in mice (Fu et al., 2019). P5091 has been reported to impair Tip60-dependent FOXP3^+^ Treg function and promote antitumor immunity in lung cancer (Wang et al., 2016). In addition, it modulated sensitivity to immunotherapy by reprogramming the TAM in lung cancer and has become a candidate drug for regulating immunotherapy (Dai et al., 2020).

Many other inhibitors have been found to inhibit USP14. It was found that VLX1570 can improve ibrutinib or bortezomib residence in Waldenstrom macroglobulinemia tumors by targeting USP14 and uchl5 and downregulating BCR-related components such as NF-κB and CXCR4 (Paulus et al., 2016). The Notch1 signaling pathway involves NF-κB and CXCR4 promoting the formation of ITAIMs. As a selective small-molecule inhibitor of USP14, IU1 can increase the expression of I-κB by reducing TNF-α, IL-1β, IL-6, and IL-8 levels (Kiprowska et al., 2017). TNF-α and IL-6 are stemness-related signaling pathway components in CSCs, and an increase in I-κB can inhibit NF-κB. In recent studies, the use of IU1 to inhibit USP14 enhanced the growth inhibition and apoptosis of breast cancer cells by enzalutamide. In addition, the combined application of enzalutamide and IU1 downregulated the Wnt/β-catenin and PI3K/AKT pathways associated with SRSs (Xia et al., 2019a). In diffuse large B-cell lymphoma, the small-molecule inhibitor of USP14 b-AP15 inhibited the spread of tumor cells by blocking the Wnt/β-catenin and TGFβ/Smad pathways (Jiang et al., 2019). b-AP15 and chemotherapeutic drugs (such as Adriamycin or VP-16) have a synergistic antitumor effect on neuroblastoma (Yu et al., 2019).

Thus, the widely studied DUB inhibitors can inhibit tumor progression and attenuate drug resistance by downregulating SRSs. The downregulation of these signaling pathways also reverses the negative impact of DUBs on TAIMs. Encouraging results have been seen in studies on other methods of DUB inhibition that regulate TAIMs (Table 3 and Figure 3). The focus has gradually shifted to how to regulate TAIMs to enhance the efficacy of immunotherapy.

**TABLE 3:** Related research on other inhibition methods of DUBs that regulate TAIM.

**Regulate TAIM**	**Inhibition methods**	**DUBs**	**Function**	**References**
Detected	KD	USP22	Increase tumor immunogenicity and tumor-infiltrating lymphocytes and improve therapeutic efficacy of CD274-targeted immunotherapy and CDDP-based chemotherapy	Huang et al., 2019
Detected	KD	USP22	Inhibit tumorgenesis and promote T cell cytotoxicity	Wang et al., 2020
Detected	KD	USP22	Improve PD-L1-targeted for anti-liver cancer immunotherapy	Huang X. et al., 2020
Potential	KD	USP22	Reverse the effects of RAS signaling and the PI3K/AKT pathway	Lim et al., 2020
Potential	KD	USP22	Reduce proliferation through down-regulating BMI1 signaling in colon cancer cells	Yuan et al., 2019
Potential	KD	USP22	Reduce the stemness and proliferation of GSCs through down-regulating BMI1 signaling	Qiu et al., 2020
Potential	KD	USP22	Decrease proliferation, migration, and invasiveness of GC cells through c-Myc/NAMPT/SIRT1-dependent signaling	Liu H. et al., 2019
Potential	KO	USP22	Down regulate c-Myc signaling and AKT and ERK pathways	Zhang K. et al., 2019
Detected	KO	USP22	Down regulate FOXP3 signaling in Treg cells	Cortez et al., 2020
Detected	KD	USP22	Reverse tumor development caused by immunosuppression	Han et al., 2020
Detected	KO	USP22	Promote the infiltration of T cells and NK cells	Li J. et al., 2020
Potential	KO	USP7	Stabilize IκBα and blocking the NF-κB pathway	Yao et al., 2018
Detected	KO	USP15	Promote T cell activation *in vitro* and enhanced T cell responses to tumor challenge *in vivo*	Zou et al., 2014
Potential	KD	USP15	Down regulate TNFα- or IL-1β-triggered NF-κB activation	Zhou Q. et al., 2020
Potential	KD	USP15	Decrease TGF-β activity	Eichhorn et al., 2012
Potential	KD	USP15	Down regulate BMI1 expression	Zhang L. et al., 2020
Detected	KO	USP15	Improve the response of melanoma transplanted mice to immunotherapy	Chen L.L. et al., 2020

*KD, gene knockdown; KO, gene knockout.*

## The Structure of USPs and the Binding Pattern and Development of Inhibitors

To date, about 100 DUBs have been identified and can be divided into different subclasses according to their protease domains. USPs, ubiquitin C-terminal hydrolases (UCHs), ovarian tumor proteases (OTUs), Machado Joseph disease proteases (MJDs), Mindys and ZUP1 are cysteine proteases, JAB1/MPN/Mov34 (JAMMs) are zinc-dependent metalloprotease (Clague et al., 2019).

Although DUB is an attractive target, the clinical development of small molecule inhibitors is limited by several obstacles. Most DUBs have an active site for catalytic cysteine, through which inhibitors can function. However, it is challenging to screen selective and effective small molecule inhibitors in DUBs with conserved catalytic pockets. In addition, the oxidative hydrolysis of cysteine at the active site will cause a high false positives in screening inhibitors (Harrigan et al., 2018). The mechanisms of DUB enzymatic activity are complex, including the use of allosteric effect or substrate-mediated catalysis, and the switching between active and inactive conformations, which make the development and selection of specific DUB inhibitors complex (D’Arcy et al., 2015). Finally, DUBs with larger molecular weight contain many domains in addition to the catalytic site. These domains can promote the recognition and connection of substrate and Ub, and regulate enzymatic kinetics. Different DUB families have divergent folds in their catalytic domain (Schauer et al., 2020b).

USPs, as the largest family in DUBs, contain three conserved domains (Cys, His, and ASP/ASN boxes), which are responsible for the reorganization of ubiquitin regulated molecules (Pfoh et al., 2015). These three domains form the binding pocket of ubiquitin and insert its C-terminal tail into the cleft between domains. At the end of the cleft, there is a cysteine triad which can catalyze the hydrolysis of peptide bond. Finally, there are two “blocking rings” (BL1 and BL2) near the binding sites of ubiquitin that can change conformation and orientation of active sites depending on substrate (Schauer et al., 2020b). Therefore, the development of most inhibitors utilizes the structural plasticity of BL1 and BL2 rings to induce the conformational changes in Ub bound DUB structures (Mevissen and Komander, 2017).

In addition, the DUBs interface in USP family is extensive, and there may be some allosteric sites in the enzyme to change the balance of ubiquitin binding. For example, the C-terminus of USP7 can directly bind to the USP domain and affect the activity (Pozhidaeva and Bezsonova, 2019). The catalytic domain of USP14 has the same structure as USP7 (Wertz and Murray, 2019). However, development of most other DUBs inhibitors focuses on compounds, which form reversible or irreversible covalent adducts with DUB catalytic cysteine. The highly reactive parts of some of these compounds may limit the drug selectivity and be toxic to patients (Kemp, 2016).

## Conclusion

In recent years, with the deepening of research, CSCs have been found to evade being killed by immune cells by changing TAMs, especially TAIMs, which hinder immunotherapy. CSCs are the key components in refractory tumor recurrence after treatment. During initial treatment, immunotherapy that leverages the body’s immune cells is the most promising way to eliminate CSCs completely. Therefore, reversing ITAIMs to enhance the ability of immune cells to kill tumor cells, including CSCs, provides new ideas for antitumor therapy. The mechanisms and the efficacy of co-application with other drugs are valuable directions of research. However, many challenges still need to be addressed.

At present, researchers have separately studied the effects of DUBs and CSCs on the ITAIM, but there is no reliable research to establish a cascade among the three. Although DUBs have been clearly confirmed to affect CSCs, there is no research on DUBs regulate TAIM through CSCs. Obviously, the establishment of a connection between the three is a new and potential perspective for tumor immunotherapy. In this review, we summarized DUBs related to these SRSs and stem cell factors, focusing on their effects on tumor development by upregulating these signals. Therefore, we searched for all the potential DUBs that could affect the SRSs, including some articles that did not prove the direct effect of DUBs on CSCs. Nonetheless, cancer cells have the dynamic ability of bidirectional conversion from non-CSC state to CSC-state (Li and Laterra, 2012). Any modification or loss of controllable differentiation can result in cancer cells with stem cell-like characteristics, such as self-renewal capability, epithelial mesenchymal transition and chemotherapy resistance (Marjanovic et al., 2013). The process is also termed phenotypic switch of CSC. Among which the regulation of the ubiquitin–proteasome system plays a significant role (Okita et al., 2007). Therefore, CSCs and cancer cells are transformed into each other in TME. However, as a potential indirect mechanism, it is impossible to determine whether cells compensate through other pathways after inhibiting DUB. The complex interactions during DUB inhibition are currently unclear. Second, because of the complexity and variability of DUB domains, the development of DUB inhibitors is extremely difficult. Only a few inhibitors of DUBs have been developed, and inhibitors of DUBs such as USP22 and USP15 have not yet led to breakthrough treatments. Moreover, although the inhibitors developed to date have shown anticancer efficacy, they lack sufficient specificity. The third point is that the current studies involving the effects of DUBs and their inhibitors on tumors are limited to preclinical trials, and the effects in clinical treatment are not clear.

In recent years, with the development of technology, single-cell sequencing (Wen and Tang, 2016; Xu et al., 2020), spatial transcriptomics sequencing (Zhou Y. et al., 2020) and ubiquitin mass spectrometry technologies (Ohtake, 2020) have matured. Accurate verification can be performed at the single-cell level, RNA level, and protein level. These technologies will be of great help in our future discovery of antitumor and immune regulatory signal pathways of DUBs. New technologies such as X-ray crystallographic analysis, virtual screening and molecular dynamics simulations have also been used to develop specific DUBs inhibitors (Li M. et al., 2020). Targeted DUBs is expected to become a new strategy to regulate TAIMs, strengthen immunotherapy, and eliminate CSCs completely to reduce tumor recurrence.

## Author Contributions

B-BC and W-LJ provided the idea and helped with the final revision of the article. J-NG, S-HD, and B-RX wrote the article. J-NG, CY, and Y-NP drew the figures and tables. All authors reviewed the manuscript and approved the final manuscript.

## Conflict of Interest

The authors declare that the research was conducted in the absence of any commercial or financial relationships that could be construed as a potential conflict of interest.

## Abbreviations

CCL: C-C motif chemokine ligand
CK2: Casein kinase 2
CSCs: Cancer stem cells
CTL: Cytotoxic T lymphocyte
CTLA-4: Cytotoxic T-lymphocyte -associated protein 4
CXCL: C-X -C motif chemokine ligand
CXCR4: C-X -C chemokine receptor type 4
DVL: Disheveled
DCs: Dendritic cells
DUBs: Deubiquitylating enzymes
EGFR: Epidermal growth factor receptor
ERK: Extracellular regulated protein kinases
FOXP3: Forkhead box P3
HH: Hedgehog
KRAS: Kirsten rat sarcoma viral oncogene
IFN: Interferon
IL: Interleukin
IRF: Interferon regulatory factor
ITAIMs: Inhibitory tumor-associated immune microenvironment
LEBF1: Lymphoid enhancer binding factor 1
MAPK: Mitogen activated protein kinase
MDSCs: Myeloid-derived suppressor cells
MHC I: Major histocompatibility complex I
mTOR: Mammalian target of rapamycin
NAMPT: Nicotinamide phosphoribosyltransferase
NF- κ B: Nuclear factor κ B
NK: Natural killer cells
PD-L1: Programmed death-ligand 1
PI3K: Phosphatidylinositol 3-kinase
PTEN: phosphatase and tensin homolog deleted on chromosome ten
S6K1: Ribosomal S6 kinase 1
SOS1: Son of sevenless 1
SRSs: Stemness-related signals
STAT3: signal transducer and activator of transcription
TAIM: Tumor-associated immune microenvironment
TAMs: Tumor-associated macrophages
TAP: Transporter associated with antigen processing
TGF- β: Transforming growth factor β
Th: T helper cell
TME: Tumor microenvironment
TNF: Tumor necrosis factor
TRAIL: TNF-related apoptosis-inducing ligand
Tregs: Regulatory T cells
Ub: Ubiquitin
USPs: Ubiquitin-specific peptidases
VEGFs: Vascular endothelial growth factors.
